# The role of KIR and HLA interactions in pregnancy complications

**DOI:** 10.1007/s00251-017-1003-9

**Published:** 2017-07-10

**Authors:** Francesco Colucci

**Affiliations:** 1 Department of Obstetrics and Gynaecology, NIHR Cambridge Biomedical Research Centre, Addenbrooke’s Hospital, University of Cambridge School of Clinical Medicine, Box 111, Hills Road, Cambridge, CB2 0SP UK; 2 Centre for Trophoblast Research, University of Cambridge, Physiology Building, Downing Street, Cambridge, CB2 3EG UK

**Keywords:** NK cells, Trophoblast, Reproduction, Diversity

## Abstract

Combinations of *KIR* and *HLA* genes associate with pregnancy complications as well as with many other clinical scenarios. Understanding how certain *KIR* and *HLA* genes influence the biology of a disease is, however, a formidable challenge. These are the two most variable gene families in the human genome. Moreover, the biology of a disease is best understood by studying the cells of the affected tissue. Natural Killer (NK) cells express KIR and are the most abundant leukocytes in the uterus. Most of our knowledge of NK cells is based on what we have learned from cells isolated from blood, but these are different from their tissue resident counterparts, including uterine NK (uNK) cells. Reproductive immunology faces an additional challenge: Two genotypes must be considered because both maternal and foetal HLA class I molecules may influence the outcome of pregnancy, most likely through interactions with maternal KIR expressed on uNK cells. Maternal uNK cells are not spontaneously cytotoxic and instead engage in interactions with trophoblast. We hypothesise that these interactions regulate allocation of resources between the foetus and the mother and may go wrong in diseases of pregnancy.

## Introduction

More than 10% of the global burden of disease is due to pregnancy complications of mother and child (WHO 2004). Association of *KIR* and *HLA* variants with pregnancy complications, including recurrent miscarriage and the gestation-specific hypertensive disorder pre-eclampsia, suggests that these genes may have a role in these conditions. This review aims to illustrate the difficulties in drawing a coherent picture that ties together the genetic evidence with the biology of uterine NK (uNK) cells. One may intuitively expect the maternal immune system to be suppressed during pregnancy to achieve tolerance to paternal antigens. This long-held view is rooted in the misleading analogy of placental implantation with organ transplantation (Colucci and Kieckbusch 2015). Recent evidence, however, shows that the maternal immune system is not all suppressed during pregnancy, and that some responses are even strengthened (Kraus et al. 2012). For example, NK and T cell responses to influenza are enhanced in pregnant women (Kay et al. 2014). Because maternal uterine NK cells (uNK) are the most abundant lymphocytes in the decidua and because they act as killer cells in the periphery, one view is that maternal uNK cells may ‘kill’ the foetus. Although popular, this view is most likely incorrect (Moffett and Shreeve 2015). Uterine NK cells, as well as other tissue resident NK cells, are poorly cytotoxic and only directly contact the trophoblast (immature placenta), not the foetus. Nevertheless, one of the first questions arising when one thinks of the immunogenetics of reproduction is almost inevitably: ‘Why does the mother not reject the foetus?’This is indeed an extraordinary paradox because like cancer cells, the foetal trophoblast cells invade deep into maternal uterine tissue, which is half-mismatched to the foetus and full of maternal NK cells (normally known for their propensity to kill cancer cells). But maternal uNK cells are not spontaneously cytotoxic and instead engage in a molecular conversation with trophoblast. Our hypothesis is that this interaction is not a conflict but a compromise that allows allocation of resources between the foetus and the mother and somehow goes wrong in diseases of pregnancy (Moffett and Colucci 2015).

**Fig. 1:**
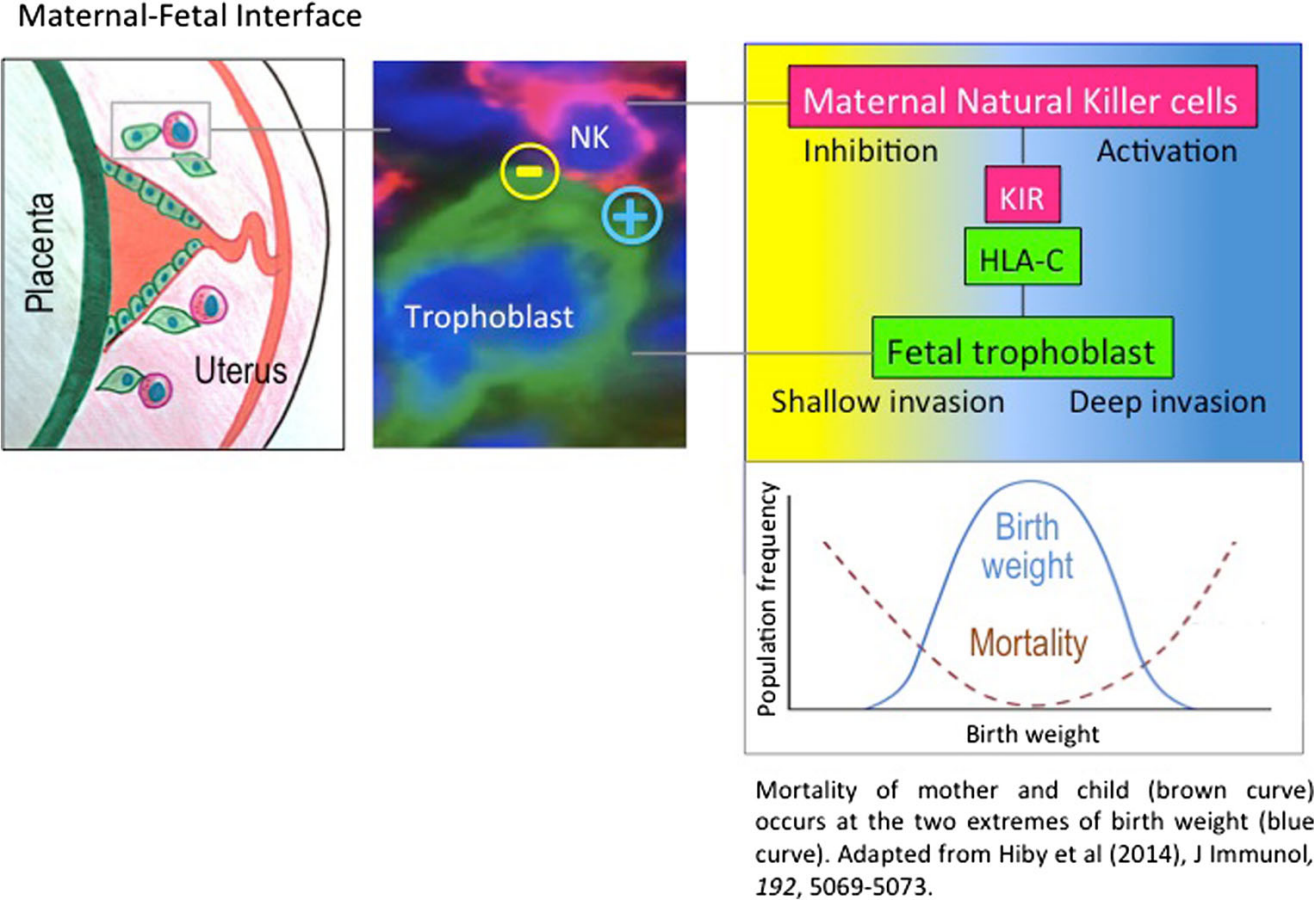
Interactions between uNK cells and trophoblast regulate allocation of resources at the maternal-foetal interface. Extra-Villous Trophoblast (*EVT, green*) transforms the uterine arteries into large conduits. Maternal uNK cells regulate trophoblast invasion. Inhibitory and activating KIR, in combination with HLA-C variants on the EVT, regulates uNK cell functions. Too much inhibition (*yellow area*) correlates with shallow trophoblast invasion and low birth weight, whereas too much activation (*blue area*) correlates with high birth weight. Mortality of mother and child (*brown curve*) occurs at the two extremes of birth weight (*blue curve*). Adapted from Hiby et al. (2014), J Immunol, *192*, 5069–5073

At the maternal-foetal boundary, trophoblast invades into maternal arteries to transform them into high conductance vessels (Fig. 1). This arterial transformation by trophoblast is essential for normal foetal growth. Trophoblast is inherently highly invasive and is regulated by uNK cells. Only extravillous trophoblast (EVT) express selective HLA class I molecules, with all other trophoblast cell types being devoid of HLA expression. EVT differentiate from trophoblast progenitors, invade deep into the uterine wall and interact with maternal immune cells, including uNK cells and monocytes. EVT express a maternal and a paternal HLA-C allotype (King et al. 2000) as well as HLA-E, HLA-G (Apps et al. 2008, 2009) and possibly HLA-F (Hackmon et al. 2017), but not polymorphic HLA-A nor HLA-B. HLA-C is, therefore, the only highly polymorphic HLA molecule expressed by trophoblast. Inhibitory and activating KIR on uNK cells bind to HLA-C on foetal trophoblast cells. Together with intrauterine foetal growth restriction (FGR), preterm labour and late spontaneous abortion, pre-eclampsia forms part of the great obstetrical syndromes (GOS), which are characterised by failure of placentation (Brosens et al. 2011). Based on genetic studies and more recent evidence in mouse models (reviewed in (Moffett and Colucci 2014), we hypothesise that excessive inhibition of uNK cells impedes trophoblast invasion leading to reduced blood flow. This ultimately reduces oxygen and nutrient delivery to the foetus and serves as the catalyst for placental stress, pre-eclampsia and low birth weight. At the opposite end of the spectrum, strong activation of uNK cells leads to macrosomia, obstructed labour and post-partum haemorrhage (Moffett and Colucci 2015), where most maternal and neonatal mortality occurrence is at the two extremes of birth weight (Fig. 1). How does this tie in with the role of KIR and HLA in NK cell biology?

Combinations of KIR and HLA genes associate with pregnancy complications as well as many other clinical scenarios including cell transplantation, HIV progression to AIDS, resolution of HCV and some malignancies (Beziat et al. 2017). Understanding how certain KIR and HLA genes influence the biology of a disease is, however, a formidable challenge. These are the two most variable gene families in the human genome. Perhaps not surprisingly, and like for other disease associations with KIR and HLA genes, there is a lack of consensus, and some reports are even conflicting. Several reasons may explain the controversies, and many have been discussed before (Khakoo and Carrington 2006; Hammond et al. 2016), including some inherent to the complexity of the KIR-HLA system and its extreme variability across individuals and populations. Other factors contributing to the lack of a coherent picture may relate to differences in sample sizes, populations studied or methodologies. Moreover, the biology of a disease is best understood by studying the cells of the affected tissue. Most of our knowledge of NK cells is based on what we have learned from cells isolated from blood. It is now clear that blood NK cells are different from their tissue resident counterparts (Bjorkstrom et al. 2016), including those found in the uterine decidua (Koopman et al. 2003). Reproductive immunology faces an additional challenge; two genotypes must be considered because both maternal and foetal HLA may influence the outcome of pregnancy, most likely through interactions with maternal KIR expressed on uNK cells.

## Generation of compound KIR and HLA diversity

**Fig. 2:**
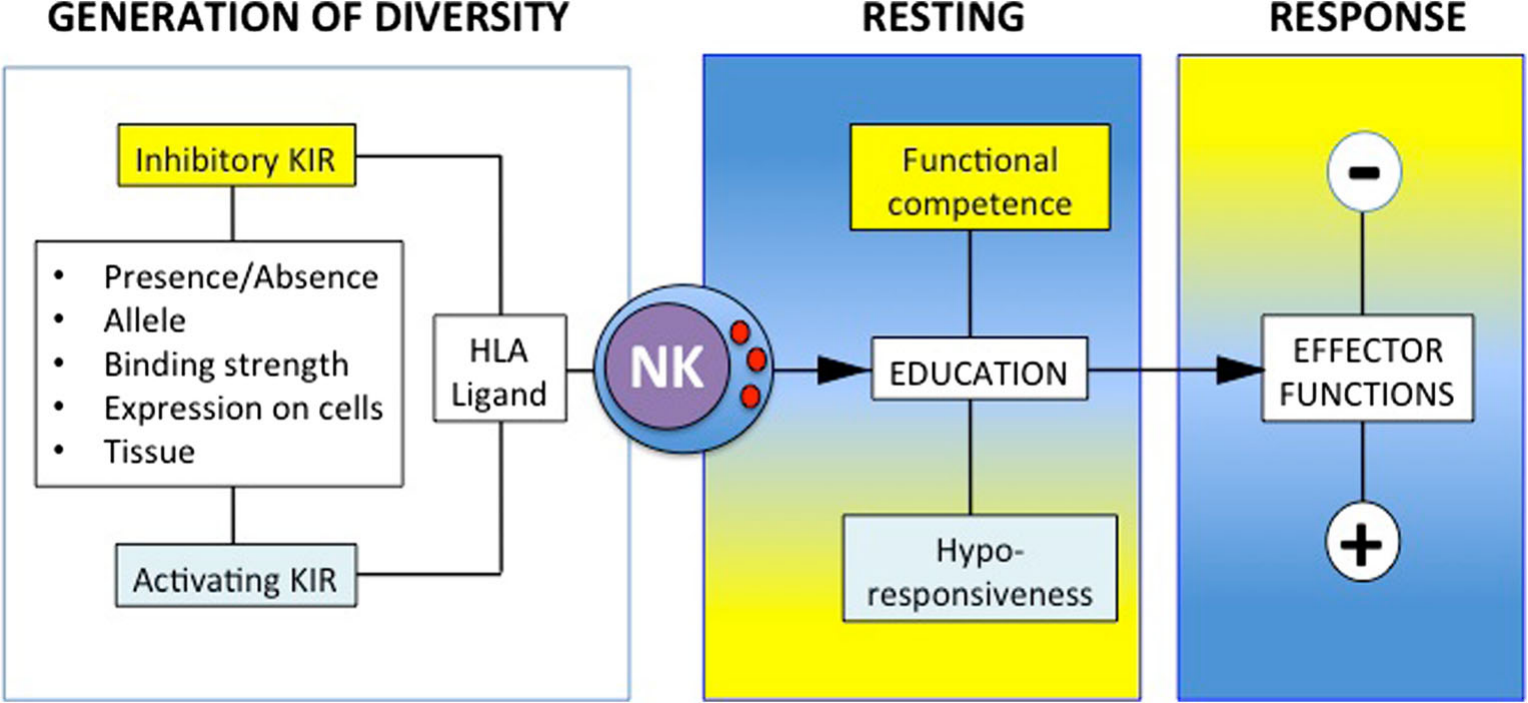
How do diverse KIR and HLA interactions influence NK cell functions? Combination of *KIR* and *HLA* variants generate diversity at many levels. Interactions of inhibitory KIR with their ligands in resting state educate NK cells. Counter-intuitively, inhibitory KIR prime functional competence, while activating KIR make cells hyporesponsive. During an immune response, or at the maternal-foetal interface, inhibitory KIR may suppress, and activating KIR elicit NK cell functions

KIR genes encode for either inhibitory or activating receptors expressed on NK cells and small subsets of T cells. The excellent review by Beziat et al., includes more information (Beziat et al. 2017). KIR diversity is generated at many levels. Due to their head-to-tail positioning on the KIR locus (within the leukocyte receptor complex, LRC, on 19q13.4), and the sequence similarity, KIRs are prone to undergo non-allelic homologous recombination, which generates diversity by contraction and expansion of the KIR locus (Traherne et al. 2010). This probably explains why KIR haplotypes are polygenic. In addition, each KIR gene has roughly 5 to 50 alleles. The synergy between haplotype and allelic diversity makes it unlikely for unrelated individuals to share the same KIR genotype. KIR haplotypes can be broadly grouped into *A* and *B* haplotypes according to their gene content. Almost all haplotypes share framework genes *KIR3DL3*, *KIR3DP1* (a pseudogene), *KIR2DL4* and *KIR3DL2*. *KIR2DL4* is a peculiar KIR because it has one inhibitory motif in its intracellular tail, but, unique among the ‘L’ KIR, it can also pair with the FcγR adaptor to activate tyrosine kinases and trigger NK cell activation (Kikuchi-Maki et al. 2005). KIR2DL4 is not expressed on the surface of peripheral NK cells but the gene is conserved between humans and primates, and the receptor is thought to be found within all NK cells. There are also null alleles of *KIR2DL4.* KIR *A* haplotypes have a fixed number of seven genes, with two activating but often non-functional KIR (*KIR2DL4* and *KIR2DS4*). Eighty percent of Caucasians have a mutation that prevents KIR2DS4 functions. Thus, most Caucasians with a KIR *AA* genotype have no activating KIR on the surface of NK cells. KIR *B* haplotypes have a variable number of genes, from four to 15 and usually at least two or more activating, functional receptors (Jiang et al. 2012). Activating receptors on the KIR *B* include *KIR2DS1*, *KIR2DS2*, *﻿KIR2DS3*, *﻿KIR2DS5* and *KIR3DS1*. Due to the presence of recombination hot spot, a centromeric (*cen*) and a telomeric (*tel*) end of the KIR locus are identifiable, with various combinations of *cen-A*, *cen-B*, *tel-A* and *tel-B* parts of the haplotype. For convention, KIR haplotypes made by *cen-A* and *tel-A* are defined as KIR *A* haplotype, whereas the combinations of *cen-A/tel-B* and *cen-B/tel-A* are defined as KIR *B* haplotype along with *cen-B/tel-B*. Despite variations in frequencies across populations, both haplotypes are found in all populations, suggesting balancing selection (Parham and Moffett 2013). Indeed, certain KIR and HLA variants may have been selected under the pressure of both pathogens and reproduction (Penman et al. 2016). Variable *KIR* gene content, allelic polymorphism and cell surface expression of both KIR and HLA individual alleles, as well as different KIR-HLA binding strengths and intracellular KIR signal strength coalesce to generate extreme diversity (Fig. 2). In addition, KIR are expressed by subsets of NK cells, and the variegated expression makes it difficult to infer the biological significance of association between *KIR* and diseases. Furthermore, the strong linkage disequilibrium among some *KIR* (i.e. *KIR2DL2* and *KIR2DS2* or *KIR2DS1* and *KIR3DS1*) makes it hard to isolate the role of individual KIRs.

Although KIR and HLA genes segregate independently on chromosomes 19 and 6, respectively, KIR expressed on NK cells bind polymorphic HLA class I molecules and their interactions regulate NK cell biology (Parham and Moffett 2013). The specificity of KIR binding to HLA class I molecules is determined by residues 76–83 within the α1 helix of the HLA class I heavy chain. These residues make up four epitopes recognized by KIR. HLA-C allotypes carry either the C1 or the C2 epitope and all HLA-C allotypes are KIR ligands. Only subsets of HLA-A and HLA-B allotypes, however, bind to KIR. The HLA-A*03 and HLA-A*11 allotypes carry the A3/11 epitope and some HLA-A and HLA-B allotypes carry the Bw4 epitope. In addition, some HLA-B allotypes carry the C1 epitopes. These four epitopes bind different subsets of inhibitory KIR. Among inhibitory receptors, KIR2DL1 binds the C2 epitope, KIR2DL2/3 binds the C1 epitope, and KIR3DL1 binds the Bw4 epitope. Among activating receptors, KIR2DS1 binds the C2 epitope, and KIR3DS1 binds the Bw4 epitope. The binding strength of inhibitory receptors is superior to that of activating receptors to the same epitope. Binding to the A3/11 epitope appears to be dependent on peptides. Unusual ligands are HLA-G, which is bound by KIR2DL4, and open conformers of HLA-F, which seem to be bound by KIR3DS1. NK cells are sensitive to the levels of HLA ligands (Hilton et al. 2012; Moesta et al. 2008), and both individual KIR and HLA alleles show differential levels of expression (Thomas et al. 2009), and these interactions may contribute to disease associations differently (Beziat et al. 2017).

## KIR and HLA influence NK cells

The interactions between KIR and HLA class I molecules regulate NK cells in at least two ways (Fig. 2). One is straightforward and operates during an immune response when inhibitory KIR suppress, and activating KIR elicit, cytokine production and/or cytotoxicity by subsets of NK cells. During a response, the lack of engagement of inhibitory KIR by the cognate HLA ligands facilitates NK cell activation through “missing-self’ recognition, that is when HLA class I molecules are downregulated following a viral infection or tumour transformation. The other way KIR and HLA interactions influence NK cells is through NK cell education, which occurs during NK cell resting state and throughout the life of an individual (Fig. 2). NK cell education is thought to be the result of the continuous KIR and HLA interactions, away from an immune response and modifiable by the tissue microenvironment. Although the process is not fully understood molecularly, its outcome is quantifiable and predictable and based on the individual’s inherited *KIR* and *HLA* variants. For example, the subset of KIR2DL1+ NK cells in an individual carrying a C2+ HLA-C allotype will respond more vigorously in assays that measure either cell cytotoxicity (typically killing of K562 erythroleukaemia targets), degranulation (by flow cytometry assessment of CD107a expression) or cytokine production (IFN-γ, MIP-1α, GM-CSF) upon NK cell activation through either co-incubation with targets or crosslinking of activating receptors. These assays may not reflect the true—and indeed poorly understood—in vivo functions of uNK cells; however, they are useful as a ‘proxy’ to quantify uNK cell activation (Sharkey et al. 2015). Although the ligands for most activating KIR are unknown, activating KIR2DS1 also binds C2+ HLA-C allotypes. The continuous interactions between KIR2DS1 and self C2+ HLA-C molecules in resting state, however, leads to hypo responsiveness, so that the subset of KIR2DS1+ NK cells will be educated to be anergic in individuals who carry C2+ HLA-C allotypes, perhaps to avoid autoimmunity (Fig. 2). Other receptors influence NK cells. For example, the conserved and non-variable CD94/NKG2A inhibitory receptor can both educate NK cells and suppress their functions upon binding to the non-classical class I HLA-E molecules. NK cell education is thought to form the basis of NK cell tolerance and to shape the repertoire of NK cells. Exposure of NK cells to cytokines produced during an immune response may however activate all NK cell subsets, educated or not, making it hard to understand the exact role of NK cell education in pathophysiology.

## Role of KIR and HLA during immune responses and in resting state (NK cell education)

The most parsimonious model to infer the role of KIR and HLA interactions from disease association studies relates to NK cell responses rather than NK cell education. In other words, and put simply, KIR *A* haplotypes are mostly inhibitory and KIR *B* haplotypes are mostly activating. The association of a condition with KIR *A* haplotype or with a given inhibitory KIR indicates that excessive inhibition of NK cells contributes to the pathogenesis of the disease. For example, the strongly inhibiting *KIR2DL2* and its C1+ HLA-C ligands were overrepresented in a cohort of 685 patients with persistent hepatitis C (HCV) infection, as compared with 352 individuals who resolved the infection (Khakoo et al. 2004). The latter instead had an increased frequency of the weakly inhibiting *KIR2DL3*. The finding suggested that weak inhibition of NK cells in the liver leads to beneficial immune responses and the resolution of infection. On the other hand, strong inhibition of NK cells may prevent immunopathology. In line with this, *KIR2DL3* may predispose to lethal cerebral malaria, presumably because it does not sufficiently refrain immune responses against the pathogen (Hirayasu et al. 2012). At the other end of the spectrum, activating KIR are associated with disease resistance. For example, *KIR3DS1* is associated with both slower progression to AIDS (Martin et al. 2002) and clearance of HCV (Khakoo et al. 2004). Donor KIR *B* haplotype is associated with better outcome following haploidentical haematopoietic stem cell transplantation in patients with acute myeloid leukaemia (Cooley et al. 2009).

Although the biological role of NK cell education is not fully understood, it is compatible with the interpretation of some association studies. The idea is that interactions between strong inhibitory KIR and self HLA class I molecules in resting state shape a functionally competent NK cell repertoire capable of responding to viral infections that cause downmodulation of the same HLA class I molecules. Examples of inhibitory receptors implicated in disease resistance are the association of KIR3DL1 and Bw4+ allotypes with slow AIDS progression and lower viral load in a cohort of over 1500 HIV+ individuals (Martin et al. 2007). This finding is in apparent contradiction with the one described earlier of association between activating KIR3DS1 and resistance to progression to AIDS. The model proposed suggests that interactions between inhibitory KIR3DL1 and Bw4+ allotypes during resting state shape a repertoire of NK cell subsets capable of springing into vigorous action when, during infections, the inhibition is lost due to down regulation of MHC class I molecules. At the other end of the spectrum of NK cell education, we find examples of disease associations with activating KIR. For example, in haematopoietic stem cell transplantation, the presence of an activating donor KIR, mostly KIR2DS1, correlates with better outcome (Cooley et al. 2009). However, if the donor also carries a C2+ HLA-C allotype, the beneficial effect of KIR2DS1 is lost, and this is thought to be due to induction of hyporesponsiveness in the KIR2DS1+ NK cell subset (Pittari et al. 2013).

## KIR and HLA association with recurrent miscarriage

Recurrent Miscarriage (RM), also referred to as recurrent spontaneous abortion or recurrent pregnancy loss, is the occurrence of three or more consecutive miscarriages with no live birth (Rai and Regan 2006). About 1% couples experience RM, with the majority of cases being unexplained and some possibly due to immune aetiology (Jauniaux et al. 2006). The lack of HLA-A and HLA-B on EVT makes it unlikely that they interact with maternal T cells. There is no evidence that human T cells reject the foetus, although elegant studies in transgenic mouse models have shown the importance of regulatory T cells in preventing abortion (reviewed in (Ruocco et al. 2014), and regulatory T cells have also been described in HLA-C mismatched human pregnancies (Tilburgs et al. 2009). Because of the variability of KIR and HLA genes, every pregnancy will have a unique KIR and HLA-C combination. This combined with the fact that uNK cells interact directly with trophoblast, suggests that KIR and HLA may play a role in RM. Studies of association between KIR and HLA variants and RM are particularly conflicting. Some of the initial studies reported associations of RM with reduced frequency of inhibitory KIR (Varla-Leftherioti et al. 2003; Flores et al. 2007), or with increased frequency of activating KIR (Vargas et al. 2009; Ozturk et al. 2012) or no association at all (Witt et al. 2004). Assessing association with KIR genes only is not sufficient. Maternal KIR and HLA class I variants, as well as paternal HLA-C variants, must be considered together to appreciate the full complexity of the system (Chazara et al. 2011). Wang found association with activating *KIR2DS1* in 73 RM patients within the Chinese Han population (Wang et al. 2007). They also found decreased frequency of C2 + HLA-C allotypes in affected patients compared to controls (Wang et al. 2007). Because C2 + HLA-C allotypes are expected to bind KIR2DS1, it is difficult to draw a coherent picture on the possible role of activating KIR2DS1 when the ligand is missing. Faridi and Agrawal found increased frequency of *KIR2DS2* combined with C1 + HLA-C homozygosity in both partners in a cohort of 177 North Indian RM patients. In addition, they also found decreased frequency of maternal *KIR2DL1* as well as C2 + HLA-C homozygosity in both partners in the RM group, suggesting excessive activation of uNK cells may contribute to RM (Faridi and Agrawal 2011).

Hiby et al. found that *KIR2DS1* was underrepresented in 95 RM patients, and instead, the KIR *AA* genotype was more frequent in the female partners of the affected couples (Hiby et al. 2008). The finding that KIR *AA* genotypes were at the expected frequency in the male partners of the affected couples was an informative internal control in this study. In addition, the frequency of C2 + HLA-C allotypes was increased in both parents in the RM couples (Hiby et al. 2008). In accordance with this, Alecsandru et al. found association with KIR *AA* haplotype in 1304 IVF treatment cycles in 291 women who experienced RM or recurrent implantation failure (Alecsandru et al. 2014). However, HLA-C was not analysed in this study. Although Dambaeva et al. did not confirm the association of RM with increased KIR *AA* genotype or decreased *KIR2DS1* frequency, they did find increased C2 + HLA-C partners among the RM cases with *KIR AA* genotype (Dambaeva et al. 2016). The scenario that emerges from these three studies suggests that excessive inhibition of uNK cells may be detrimental. In the study by Dambaeva et al., it was also reported that C2 + HLA-C alleles were more frequent within the *KIR2DS1*+ female partners of the RM couples (Dambaeva et al. 2016). One interpretation is that NK cell education may make the KIR2DS1+ decidual subset hyporesponsive, thus preventing its potentially beneficial contribution to maintenance of pregnancy. This would be consistent with a role for uNK cell education in preventing RM. As initially conceived, the role of NK cell education through inhbitory KIR may be to prime NK cells in resting state to spring into action during MHC downregulation (missing–self recognition). Missing-self recognition at the maternal-foetal border, however, never occurs in pregnancy because EVT always express one of the maternal HLA haplotype. Moreover, >90% uNK cells express the inhibitory NKG2A receptors that bind HLA-E on trophoblast. Therefore, if and how inhibition during resting state (NK cell education) benefits uNK cell effector functions during pregnancy is unknown. It is hard to directly compare these studies on KIR and HLA association with RM because they were done in different populations, and using different methodologies (Moffett and Hiby 2009).

## KIR and HLA association with pre-eclampsia

Pre-eclampsia is an enigmatic and understudied disease that only occurs in pregnancy and leads to preventable maternal and perinatal deaths, particularly in Sub-Saharan Africa, where the prevalence reaches 8–10% (Nakimuli et al. 2014). Pre-eclampsia arises from a primary defect in placentation leading to placental stress, triggering systemic decompensation of endothelial cells. The syndrome is characterized by a range of clinical signs including high blood pressure, proteinuria and edema (Redman and Sargent 2005). The UK National Health Service estimates that around 1000 children die each year in the UK because of pre-eclampsia (NHS. Pre-eclampsia 2015). Pre-eclampsia occurs more often in first pregnancies, with an incidence of 4.1% in nulliparous women decreasing to 1.7% in subsequent pregnancies. However, a history of pre-eclampsia markedly predisposes to subsequent pregnancy complications (van Oostwaard et al. 2015), with a risk of recurrence of about 15 and 30 times higher after one or two pregnancies with pre-eclampsia, respectively. This risk reaches 30 and 60 after one or two pregnancies with severe, early onset pre-eclampsia (Hernandez-Diaz et al. 2009). These epidemiological and clinical data are consistent with two disease entities: a sporadic, milder form of pre-eclampsia accounting for ~1% of all pregnancies and a recurrent, more serious condition, accounting for 50–75% of cases in primiparous women. While the sporadic type may be due to transient factors, such as twin pregnancies, the latter has more familial aggregation, causes preterm birth and other maternal and foetal morbidities, and it is probably affected by environmental and genetic factors (Hernandez-Diaz et al. 2009). Pre-eclampsia clusters within families and studies of concordance in pre-eclamptic monozygotic twins suggest a genetic basis for the pathogenesis of the disease (Chesley et al. 1968; O'Shaughnessy et al. 2000). This, together with the observation of recurrent pre-eclampsia, suggest the involvement of maternal genetic factors (Klungsoyr et al. 2012). The key role of the placenta in the pathogenesis indicates that foetal genes may also contribute. Indeed, genetic factors contribute to more than half the susceptibility to develop pre-eclampsia. Maternal, foetal and couple genetics explained the variance in susceptibility to pre-eclampsia (Cnattingius et al. 2004). Uniquely among human diseases, therefore, interactions between maternal and foetal genotypes are important, and this poses both challenges and opportunities in understanding the pathogenesis of pre-eclampsia.

Association of KIR and HLA with pre-eclampsia is perhaps less controversial than with RM. Although an initial study focussing on only one KIR gene, *KIR2DL4*, found no association (Witt et al. 2002), Hiby et al., found that maternal KIR *AA* genotypes and foetal C2 + HLA-C allotypes were more frequent in the pre-eclampsia group. Subsequent studies by the Moffett lab confirmed that the *KIR AA* genotype is overrepresented in pre-eclamptic pregnancies carrying C2 foetuses in an extended UK cohort and in an independent Ugandan cohort (Hiby et al. 2004, 2010; Nakimuli et al. 2015). Of note, C2 + HLA-C allotypes are more frequent in Africans (Beziat et al. 2017). Analysis of the parental origin of C2 indicated the paternal HLA-C confers the risk (Hiby et al. 2010). This was later substantiated by experiments showing that the important receptors may be KIR2DL1 on the *A* haplotype and KIR2DS1 on *B* haplotypes, because both bind to C2+ HLA-C allotypes, but one suppresses and the other elicits uNK cell responses that may benefit placentation (Xiong et al. 2013). Importantly, both KIR2DL1 and KIR2DS1 are expressed by large subsets of uNK cells (Sharkey et al. 2008). One explanation for the observed risk association with KIR *AA* and C2 is the greater number of inhibitory KIR and the lack of activating KIR on the KIR *A* haplotype. Moreover, a similar association was found with FGR (Hiby et al. 2010) and birth weight (Hiby et al. 2014). Evidence from mouse studies also suggests that exaggerated uNK inhibition may be one key mechanism underlying pregnancy complications (Kieckbusch et al. 2014). On the other hand, haplotype *B* with KIR2DS1 is underrepresented in pre-eclamptic pregnancies with C2+ HLA-C foetuses and in pregnancies with high birth weight, suggesting that active uNK cells may prevent pre-eclampsia and drive foetal growth. *KIR2DS4*, present on *tel-A* in Caucasians, and alleles of *KIR2DS5*, present on *cen-B* in Africans, are also associated with resistance to pre-eclampsia (Nakimuli et al. 2014; Kennedy et al. 2016). These findings corroborate the notion that activation of uNK cells enhances trophoblast invasion, and leads to foetal growth (Moffett and Colucci 2014). Another scenario, compatible with association of uNK cell education with disease resistance emerged (Parham and Guethlein 2010). Within the *KIR AA* pregnancies with C2+ HLA-C foetuses, women homozygous for C1 *HLA-C* were more at risk than those carrying at least one C2 *HLA-C* allele. This suggests that maternal C2+ HLA-C allotypes educate KIR2DL1+ uNK cells in resting state and counter the inhibitory input of foetal C2+ HLA-C allotypes during pregnancy (Hiby et al. 2010). However, differences in cell surface expression, binding strength and signalling functions of individual KIR alleles, as well as functional differences among HLA-C alleles may confound the results (Beziat et al. 2017). Studies in large cohorts that allow isolating the effect of alleles, in combination with experiments that allow quantification of uNK cell functions, will help to identify high-risk alleles.

## Other interactions

Beyond the interactions between maternal KIR and foetal HLA-C, others must be considered to appreciate the full picture (Ponte et al. 1999). Foetal HLA-G on trophoblast, for example, can interact with uNK cells and monocytes through KIR2DL4 and LILRB (Rajagopalan and Long 1999). Moreover, the leader peptide of HLA-G is a powerful modifier of HLA-E expression, and as such can modulate uNK cell functions mediated by inhibitory NKG2A and activating NKG2C. HLA-F has recently gained attention, as it can activate NK cells through binding KIR3DS1 (Burian et al. 2016; Garcia-Beltran et al. 2016). While NKG2D and NKp46 may activate uNK cells, 2B4 may be an important inhibitory receptor as its signalling properties are specific to uNK cells (Vacca et al. 2006), and the ligand is present at the maternal-foetal interface (Apps et al. 2008). NKp30 and NKp44 too may acquire decidual-specific signalling properties following expression of splice variants that make them key inhibitory receptors (Siewiera et al. 2015). The interactions between maternal KIR and maternal MHC class I molecules extend beyond HLA-C, because decidual cells express HLA-A and HLA-B, and these may influence uNK cell education (Sharkey et al. 2015).

## Concluding remarks

On the wake of the recent success stories in immunology that are changing clinical practice to treat patients with autoimmunity and cancer, one may anticipate similar successes in other areas of immunology. None of the studies on RM, however, appears conclusive. The misconception that the maternal immune system, including uNK cells, must be suppressed during pregnancy, has led to the unsubstantiated view that activation of uNK cells may drive some sort of foetal rejection. The interpretation of some of the initial association studies with RM seems influenced by this view. A large cohort study in a homogeneous population, with a relevant control group and standardized typing of maternal KIR and both maternal and foetal HLA-C may shed more light on the role of KIR and HLA in RM. More robust association has been shown of certain combinations of maternal KIR and foetal HLA-C variants, particularly the paternal HLA-C, with pre-eclampsia, FGR and birth weight. Despite the compelling evidence, we are still far from clinical applications (Moffett et al. 2016). For example, the combination of the KIR *AA* genotype and foetal C2 + HLA-C is found in about 10% of pregnant Caucasian women. This combination exposes these pregnant women to a risk of pre-eclampsia twice as great as that of the general population. This is a significant risk; however, the predictive power of the association is not clinically useful. Combining this with other clinical and serological data may lead to generate algorithms that may yield a better predictive power. For example, a new and elegant mathematical model predicts birth weight based on *KIR* and *HLA* interactions (Clark et al. 2017).

We have learnt much about variability in *KIR* gene content, polymorphism, inhibitory and activating receptors, variegated expression, and we are learning about tissue-specific factors that regulate NK cells. The major challenge is to tie together the genetic data with the molecular mechanisms of disease (Beziat et al. 2017). Moreover, we do not know the ligands for many activating receptors, nor the role of trophoblast peptides in regulating uNK cells. Also, it is very hard to pinpoint how exactly KIR and HLA variants affect NK cell education and effector functions. New technology may tackle the extreme diversity of compound KIR and HLA genotypes. Refined lab assays may help to better understand the biology of NK cells. For example, we do not really know how to measure uNK cell responses in vitro, and we rely on ‘proxy’ assays that measure cytokine production and degranulation, which apply mostly to blood NK cell functions. Intelligent use of mouse models could shed some light on the immunogenetics of uNK cells and new transgenic mice expressing specific *KIR* and *HLA* variants could help to analyse the role of certain combinations in vivo. In 2005, Rajagopalan and Long wrote in a commentary (Rajagopalan and Long 2005): ‘Hopefully a molecular basis for the new disease associations with HLA– KIR gene combinations will be provided before the year 2025’. They were referring to the 20 years or so that lapsed between the first disease association with HLA polymorphism in the 1970s and the resolution of the structure of the TCR bound to the MHC molecules in the 1990s. The challenge ahead is formidable, but we may be approaching the objective and indeed, in some areas of medicine, such as haematopoietic stem cell transplantation and treatment of hepatitis, KIR typing is entering the clinical practice and may improve patient outcome (Foley et al. 2014; Vidal-Castineira et al. 2014; Stelma et al. 2016).
